# A Preschool Rhythm and Movement Intervention: RCT Evidence for Improved Social and Behavioral Development

**DOI:** 10.3390/bs16010100

**Published:** 2026-01-12

**Authors:** Kate E. Williams, Laura Bentley

**Affiliations:** 1 School of Education and Tertiary Access, University of the Sunshine Coast, Sippy Downs, QLD 4556, Australia; 2 School of Education, Queensland University of Technology, Kelvin Grove, QLD 4059, Australia; 3 Child Health Research Centre, The University of Queensland, St Lucia, QLD 4072, Australia; laura.bentley@uq.edu.au

**Keywords:** self-regulation, early childhood, music, rhythm, intervention, RCT, behavior problems, social development

## Abstract

Active music and movement engagement has been widely integrated in human socialization across history and cultures, and is particularly prevalent in early childhood play and learning. For clinical populations, music therapy is known to support social skills and wellbeing for young children. However, there is less evidence for the value of active music engagement for non-clinical populations in terms of supporting social and behavioral wellbeing in the early years. This study reports results from the Rhythm and Movement for Self-Regulation (RAMSR) program delivered by generalist kindergarten teachers in low socioeconomic communities. This randomized control trial involved 213 children across eight preschools in disadvantaged communities in Queensland, Australia. The intervention group received 16 to 20 sessions of RAMSR over eight weeks, while the control group undertook usual preschool programs. Data was collected through teacher report at pre and post intervention, and again six months later once children had transitioned into their first year of school. Robust mixed models accounting for repeated measures and clustering of children within kindergartens (random effects), evidenced significant intervention effects across the three time points for improved prosocial skills (*p* = 0.04, *n_p_*^2^ = 0.02), and reduced externalizing (*p* < 0.01, *n_p_*^2^ = 0.03) and internalizing behavior problems (*p* = 0.04; *n_p_*^2^ = 0.02), with small to moderate effect sizes. These findings highlight the valuable role that intentional active music engagement in universal settings such as preschool can play in terms of social and behavioral wellbeing. The importance of these results lies in the fact that children from lower socioeconomic backgrounds are more likely to experience risks to social and behavioral development, requiring additional supports, yet experience inequities in access to high-quality music and movement programs.

## 1. Introduction

Music and movement engagement have been a key feature of human socialization and behavioral regulation across history and cultures (McPherson & Welch, 2018). In early childhood, informal music experiences and formal music training has been linked with a range of enhanced non-musical outcomes including enhanced social skills (Akyurek et al., 2025) and increased prosocial behaviors (Ilari et al., 2020). In this paper, we focus on social development, including general sociability, prosocial behaviors, and externalizing and internalizing behavior problems across the important transition-to-school period. These constructs are highly malleable in the early years and are influenced by genetic disposition (e.g., temperament), early environments (e.g., socioeconomic status and parenting; Allee-Herndon & Roberts, 2019; Blair & Raver, 2012; Finders et al., 2021), and important antecedent skills including self-regulation (Taylan & Çarşanbalı, 2025). Children experiencing socioeconomic disadvantage are at a greater risk of delays in social development (Gautam et al., 2024) and increased behavioral problems, and are also less likely to have access to high-quality early music experiences (Kennewell et al., 2022), underscoring the importance of accessible interventions. The Rhythm and Movement for Self-Regulation (RAMSR) program is the first early childhood intervention to draw specifically on neuroscientific evidence pertaining to rhythm and movement, and the discipline of music therapy, to address social skills and behavioral problems. This paper presents the findings of a RAMSR randomized control trial (RCT) in relation to social development and behavioral problems. Prior publications have reported RAMSR intervention findings related to cognitive development including self-regulation, executive function, and school readiness (Bentley et al., 2023; Williams et al., 2023).

### 1.1. Social and Behavioral Development and Socioeconomic Disadvantage

The emergence of social skills and behavior during early childhood is a major focus of developmental and clinical research. In this study, we focus on four key areas of social-emotional development: prosocial behaviors, general sociability, and internalizing and externalizing behavior problems. Prosocial skills refer to behaviors undertaken that benefit others such as helping, sharing, and cooperation (Penner et al., 2005). Stronger early childhood prosocial skills have been linked longitudinally with better executive functioning (Moriguchi et al., 2020), positive academic achievement (Collie et al., 2018), increased peer acceptance (Ettekal & Mohammadi, 2020), and enhanced self-esteem (Coulombe & Yates, 2021). Sociability refers to young children’s tendency to seek, initiate, and enjoy social interactions; display positive engagement with peers, and respond warmly and cooperatively in social contexts. Higher sociability in childhood is correlated with reduced behavior problems (Hukkelberg et al., 2019) and enhanced academic achievement (Frogner et al., 2022). Therefore, understanding effective ways to support these skills in early childhood settings has become a research area of interest.

Understanding the etiology of early behavior problems and intervening to address these is an ongoing focus in research, given developmental pathways that suggest early problems beget later problems with implications for both wellbeing and achievement outcomes (D’Souza et al., 2019; Hammerton et al., 2019). Externalizing and internalizing behavior problems often co-occur in young children, with higher early childhood externalizing behavior problems predicting later internalizing problems (Oh et al., 2020). Children with higher levels of early behavior problems are at risk of peer victimization (D’Urso & Symonds, 2022), poorer relationships with teachers, and poorer academic achievement (Shi & Ettekal, 2021). Behavior problems that remain unresolved in early adolescence significantly increase the risk of depression, alcohol use disorders, and suicide (Blain-Arcaro & Vaillancourt, 2019; Meque et al., 2019; Soto-Sanz et al., 2019).

Children from low socio-economic backgrounds are known to be at higher risk for poorer social skills and elevated behavior problems (Cuartas et al., 2022), and so are an important target group for early intervention. Importantly, boosting social skills in children from low socioeconomic backgrounds may provide a buffer against academic achievement disparities common in these communities. Stronger prosocial skills in particular, specifically the propensity to be kind and generous towards others, has been identified as a protective factor, reducing the risk of emotional problems and increasing academic achievement in low socioeconomic contexts (Armstrong-Carter et al., 2021; Perry et al., 2018).

### 1.2. Connecting Rhythm and Movement to Social and Behavioral Development

The concept of interpersonal synchrony is key to understanding why group rhythmic movement activities may be beneficial for enhancing social and behavioral development. This is generally considered to be “the temporal coordination of actions, emotions, thoughts and physiological processes” (Mayo & Gordon, 2020, p. 1). Focusing specifically on interpersonal movement synchrony, music—including beat and rhythm—is a key context in which humans move together. Rituals incorporating collective rhythmic coordination, such as chanting, dancing, singing, and drumming, have long been part of human culture, and are believed to strengthen social bonds and promote prosocial behaviors (Fischer et al., 2013). From an evolutionary perspective, group synchronized movement serves to strengthen in-group bonding (Tunçgenç & Cohen, 2016). Interestingly, moving in synchrony has been shown to enhance bonding with out-group members more than non-synchronous movement (Tunçgenç & Cohen, 2016). Additionally, moving in synchrony with others has been associated with higher self-esteem compared to moving to one’s own rhythm (Lumsden et al., 2014).

Developmentally, interpersonal synchrony has been researched in the context of early infant–caregiver dyads. As adults provide rhythmic information in their interactions with infants, including touch, singing, and movement, infants can entrain to this rhythmic data (Markova et al., 2019). This interpersonal synchrony supports attunement and reciprocal interactions between infants and caregivers (Markova et al., 2020). This physical synchronization can extend beyond motor alignment to include emotional resonance between individuals. Affective entrainment, which involves individuals sharing an emotional state, plays a key role in the formation of social bonds and can also emerge through shared, entrained movement (Leclère et al., 2014). Musical activities, ranging from simple nursey rhymes between a caregiver and child to a complex choir performance, offer opportunities for affective entrainment, further enhancing interpersonal connection (Phillips-Silver & Keller, 2012).

The beneficial role of interpersonal movement synchrony in relation to social bonding and prosocial outcomes beyond very early infant–caregiver dyads has been studied in a number of contexts. In one laboratory study, infants aged 14 months were either bounced face-to-face in time (synchronously) or out of time (asynchronously) with an adult partner (Cirelli et al., 2014). Following this experience, infants in the synchronous condition exhibited significantly more helping and prosocial behaviors with their adult partner than those in the asynchronous condition (Cirelli et al., 2014). Using a specialized swing set with pairs of four-year-olds in the US, researchers found that children swung synchronously performed better on cooperation tasks (Rabinowitch et al., 2024), and were more willing to share, even with unfamiliar peers (Rabinowitch & Meltzoff, 2017), compared to those swung asynchronously. In an English study, pairs of 4–6-year-old children faced each other and played a tapping and clapping game along to a beat provided on their own individual headphones (Tunçgenç & Cohen, 2018). Pairs were assigned to either the synchronous condition (the same tempo stimulating their clapping and tapping) or asynchronous condition (different tempos provided to each child). Children in the synchronous condition were almost 30 times more likely to be helpful to their partner in an experimental helping task following this experience, compared to their peers in the asynchronous condition. Synchronous pairs also engaged in more mutual smiling and eye contact with each other than asynchronous pairs (Tunçgenç & Cohen, 2018). These prosocial effects of joint rhythmic participation are not confined to experimental studies. In observational studies across the United States and Australia, children with higher levels of home music engagement or exposure to formal music early-learning programs have been shown to have significantly higher prosocial behavior than their peers with no or lower early music engagement experiences (Ilari et al., 2020; Williams et al., 2015).

Key to understanding the potential of rhythmic movement experiences in reducing risk of behavioral problems for children is understanding their role in boosting self-regulation and executive function, specifically through the practice of beat synchronization (also known as sensorimotor synchronization). Beat synchronization is the ability to coordinate movement in time to an external stimulus, often an auditory beat (Repp & Sue, 2013). Covariation with other observable skills suggests shared neural pathways with those related to motor and cognition. For example, poorer synchronization skills have been linked with dyslexia (Fiveash et al., 2021), attention deficit hyperactivity disorder (Slater & Tate, 2018), and lower executive functioning (Lesiuk, 2015). We have previously argued that the practice of beat synchronization (also known as sensorimotor synchronization), will support the strengthening of neural pathways associated with executive function and self-regulation (Williams et al., 2023). Given that self-regulation is a protective factor for the development of later internalizing and externalizing behavior (Robson et al., 2020), it is possible that group rhythmic movement engagement will reduce the risk of later behavior problems for young children.

### 1.3. Rhythm, Movement and Music Experiences for Social and Behavioral Development

A range of formal and informal music experiences have identified positive effects on social and behavioral development in young children. Group choral practice with children aged 7–8 years has increased prosocial skills when compared to participation in arts or competitive games (Good & Russo, 2016), and formal ukulele instruction over a period of 10 months with 8-year-olds has been associated with increased prosocial skills compared to a control group (Schellenberg et al., 2015). Overall, in regard to the benefits of formal music instruction, a range of primarily correlational studies indicate that child musicians tend to have stronger empathy and broader social–emotional skills compared to non-musicians (Wu & Lu, 2021).

Informal or play-based music experiences, which can involve musical play activities with caregivers, teacher or peers rather than learning music theory or an instrument have also been associated with positive social and behavioral development (Williams, 2018). For example, in a longitudinal correlational study, informal musical play at home with caregivers at 2–3 years old was associated with increased attentional regulation skills and enhanced prosociality two years later when children were aged 4–5 years, even when levels of shared book reading were accounted for (Williams et al., 2015). Joint music-making in classrooms with four-year-old children, involving percussion instruments, singing, and moving together in synchrony, promoted prosocial behavior, including spontaneous helping and cooperative problem-solving when compared to a non-musical activity (Kirschner & Tomasello, 2010). Children were also more likely to choose to cooperate with another partner rather than play alone, following a shared musical experience, as opposed to a shared non-musical experience, suggesting increased sociability (Kirschner & Tomasello, 2010). Similarly, preschoolers aged 3 to 5 years displayed more helping behavior toward the researchers after engaging in musical play compared to non-musical play (Beck & Rieser, 2022).

Studies of specialized music therapy programs have documented some promising social and behavioral outcomes for both non-clinical and clinical populations. For example, The Musical Contour Regulation Facilitation intervention delivered by a music therapist in preschool settings reported improved emotional regulation, lowered aggression, and fewer internalizing and externalizing problems in a small sample of children with pre- and post-intervention measures (Moore & Hanson-Abromeit, 2023). An after-school music therapy program for children in elementary school with behavioral and emotional problems also documented improvements over a 20-week period (Chong & Kim, 2010). However, neither of these studies included a control group comparison. One of the very few experimental trials of music therapy for children in relation to behavioral problems found improved self-esteem and reduced depression in the intervention group in a clinical setting, compared to a control group (Porter et al., 2017).

Taken together, while there is promising evidence demonstrating prosocial and behavioral benefits from various music, rhythm, and movement engagement, overall, the evidence base has been limited by a limited focus on early childhood, small sample sizes, non-randomized control trials, and where there is robust RCT evidence, the intervention has typically been delivered by specialists such as music therapists, and thus may not be scalable. The RAMSR program, the focus of this study, is the first, to our knowledge, to specifically apply the science of rhythmic movement engagement to a non-clinical early childhood population, enabling educators to deliver the program within everyday settings to boost social skills and reduce behavioral problems.

### 1.4. The RAMSR (Rhythm and Movement for Self-Regulation) Program and Evidence to Date

RAMSR is a result of the collaboration between music therapists, music specialists and early childhood educators who aimed to draw together evidence and practice from music psychology, formal music education, and clinical music therapy to design an intervention that can be implemented by any adult working with children aged 3 to 8 years (Williams, 2018). The program consists of four structured sessions plans which gradually increase in complexity, with each plan designed to be repeated before advancing to the next. Each plan includes six components: (1) warm-up, including body percussion activities; (2) becoming familiar, including adaptions of well-known early childhood songs; (3) moving to the beat, involving large gross motor movement activities; (4) playing to the beat, involving simple rhythmic exercises using sticks or castanets; (5) dancing to the beat, including more intricate gross motor movement sequences, often integrating visual–motor coordination; (6) calming, consisting of relaxation activities involving gentle movement followed by stillness, accompanied by calming music to support physiological entrainment (see Table 2). For each activity (a total of 24 activities across 4 session plans with 6 components), multiple extensions are available, enabling facilitators to progressively increase the level of challenge for children within each session plan.

Previous research on the RAMSR program has demonstrated positive outcomes for children experiencing socioeconomic disadvantage. In an initial quasi-experimental pilot study (*n* = 113), the program was delivered by visiting specialists, and the findings indicated improvements in teacher-reported emotional regulation across the cohort, along with gains in the executive function skill of cognitive flexibility for boys, compared to the control group (Williams & Berthelsen, 2019). A subsequent randomized control trial (*n* = 213), where preschool teachers delivered the program, demonstrated immediate benefits for teacher-reported self-regulation for the intervention group compared to the control group (Williams et al., 2023), with these effects sustained 6 months later as children transitioned to their first year of school (Bentley et al., 2023). The follow-up assessment also revealed improvements for the intervention group in school readiness and enhanced inhibition compared to the control group (Bentley et al., 2023).

### 1.5. The Current Study

The current study extends prior research by evaluating the social and emotional outcomes associated with participation in the RAMSR program. While our prior work has documented outcomes related to self-regulation and executive function (Bentley et al., 2023; Williams et al., 2023), the social and behavioral outcomes investigated here have not been previously investigated. Specifically, we focus on four indices of social and behavioral development hypothesizing that children who participate in the RAMSR intervention, compared to those who do not, will show improved prosocial skills and sociability, and reduced externalizing and internalizing behavior problems. This study contributes to an emerging body of work that investigates the role of shared rhythmic movement participation in supporting social development, and an even scarcer body of work investigating the role of such experiences in terms of behavior problems. Importantly, this study contributes through a careful RCT design with implementation of the intervention by regular, non-specialist early childhood teachers in universal settings. This is an important contribution given that scaling up any successful approaches require accessibility to the children who are in the most need of developmental support.

## 2. Materials and Methods

### 2.1. Design

This research employed a clustered randomized control trial with approval from the Queensland University of Technology Human Research Ethics Committee (approval number 1900000566), and is registered with the Australian New Zealand Clinical Trials Registry, ACTRN12619001342101.

### 2.2. Sample and Description of Participants

Full details on the study context and sample, and the intervention effects for self-regulation, are provided elsewhere (Bentley et al., 2023; Williams et al., 2023). In brief, this study involved eight kindergarten centers in Queensland, Australia (the year before formal schooling in this jurisdiction). Eight centers were randomized to achieve adequate statistical power at the cluster level while preserving a balanced and feasible design. Based on pilot-derived estimates (Williams & Berthelsen, 2019), accounting for clustering using an anticipated intraclass correlation of approximately 0.03 yielded a design effect of up to 1.9, indicating that the resulting effective sample size was sufficient to detect small intervention effects after allowing for expected consent and attrition rates.

**Figure 1:**
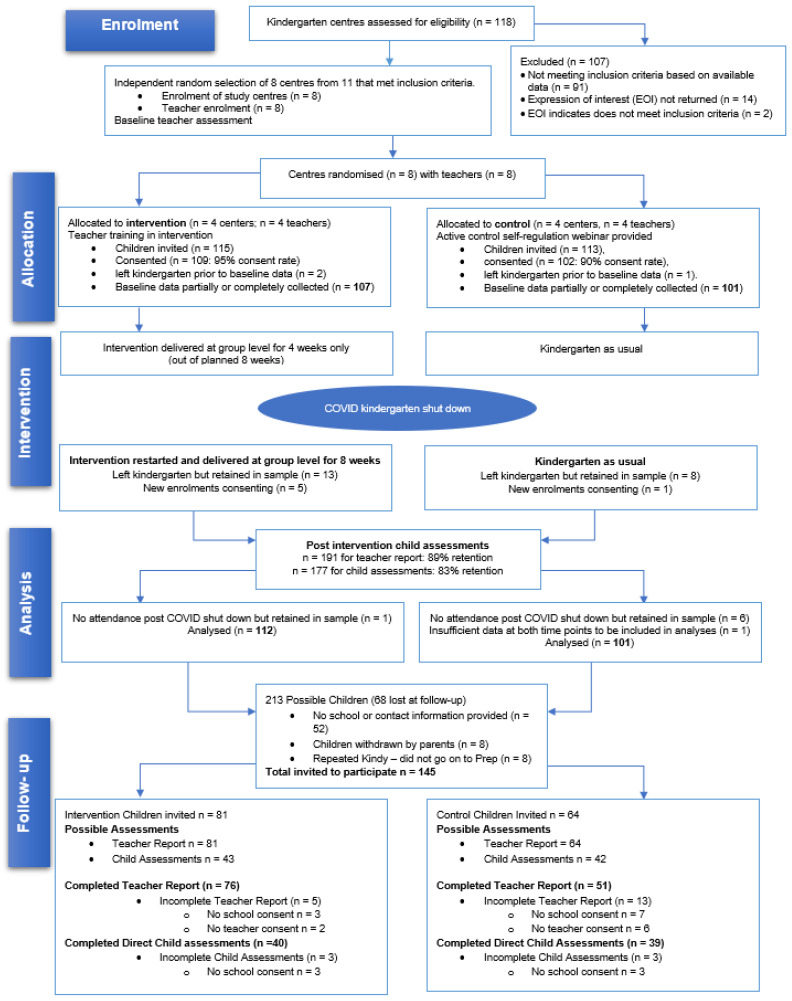
Consort flow diagram (Bentley et al., 2023).

Children typically begin their kindergarten year at approximately 3.5–4.5 attending for 12 months prior to formal schooling, with sessions scheduled for four to five days per fortnight. All participating centers were located in communities identified as having a higher-than-average level of child vulnerability in child social and emotional development (Australian Early Development Census [AEDC], 2021). At the start of 2020, 213 children commencing kindergarten at the eight centers were recruited. Baseline assessments (T1) were conducted, followed by reassessment six months later, immediately after participation in the intervention (*n* = 4 centers) or the control condition (*n* = 4 centers). A further six-month follow-up (T3) occurred after children transitioned to their first year of school, known locally as Prep. Following additional consent for T3, 79 child assessments were completed (48% female; 51% intervention) and teacher reports were obtained for 129 children (51% female; 60% intervention). See Figure 1 for the CONSORT diagram. Children retained at follow-up were representative of the original sample in terms of sociodemographic characteristics (Bentley et al., 2023). Sociodemographic details are provided in Table 1.

### 2.3. Teacher Training and Intervention Implementation Fidelity

The teachers assigned to deliver the intervention received training for the program and practiced implementation with their kindergarten cohort during the year prior to the trial (2019). In 2020, they delivered the full intervention to the newly recruited kindergarten cohort. Training consisted of a one-day workshop followed by fortnightly on-site coaching. Teachers were provided with all session plans, audio tracks, and instructional videos demonstrating the activities. The intervention was integrated into the regular kindergarten schedule, typically during circle time at the start of the day. After each session, teachers completed a checklist rating children’s attention, participation, enjoyment, and success, as well as their own enjoyment and confidence, using a three-point scale for each (low, moderate, high). Implementation fidelity was high, with an average of 92.5% adherence to the planned activities, and both child and teacher engagement were rated as high for more than 70% of sessions (see Williams et al., 2023, for full details). Teachers in the control condition were provided with a one-off webinar recording of professional learning related to children’s self-regulation development, and were provided with the full RAMSR training following completion of the study.

### 2.4. RAMSR Activities

Access to the RAMSR intervention is provided through the completion of professional learning, which is available online to all adults at https://rhythmicintegrations.com/rhythm-and-movement-for-self-regulation/ accessed on 10 January 2026. Each of the four session plans follows the same structure as shown in column 1 in Table 2. Table 2 provides explanations of some of the kinds of activities undertaken in each section of the plan.

### 2.5. Measures

Across all three time points, measures using teacher report remained consistent. Kindergarten teachers completed teacher-report measures for baseline (T1) and immediately post-intervention (T2), and Prep teachers completed these at follow-up (T3).

We used four subscales from the Child Self-Regulation and Behavior Questionnaire (CSBQ) (Howard & Melhuish, 2017) which is an educator report requiring the respondent to evaluate the general frequency of target behaviors on a scale of 1 (not true) to 5 (certainly true). The prosocial subscale consists of five items; for example, ‘happy to share’ and ‘helps others’ (α = 0.91 T1; 0.88 T2; 0.91 T3). The externalizing behavior problem subscale consists of five items; for example, ‘aggressive to children’ and ‘most days will lose temper’ (α = 0.84 T1; 0.86 T2; 0.88 T3). The internalizing behavior problem subscale consists of five items; for example, ‘most days distressed or anxious’ and ‘most days says feeling unwell’ (α = 0.78 T1; 0.77 T2; 0.68 T3). The sociability subscale consists of seven items; for example, ‘chosen as a friend by others’ and ‘plays easily with other children’ (α = 0.90 T1; 0.90 T2; 0.91 T3).

To support meaningful longitudinal comparisons, scores were transformed using the proportion of maximum scaling (POMS) approach rather than z-standardization. POMS places measures with differing response formats on a common metric while preserving the original distributional properties of the data, avoiding the confounded frame of reference and consequential interpretive distortions that can arise when standardization is applied to repeated measures over time (Moeller, 2015). POMS transformed scores ranged from 0 to 1, where a higher score related to a higher raw score.. The covariates used in analyses were reported by parents and included child gender (1 = female; 0 = male); Aboriginal and/or Torres Strait Islander Status (1= yes; 0 = no); English not the primary language at home (1 = yes; 0 = no); family income (low-income [<$1000 USD/wk] = 1; not low-income = 0); parent education achieved (1 = did not complete high school; 0 = complete high school; and parental concerns for any child developmental delay (1= yes; 0 = no).

### 2.6. Approach to Analysis

To ascertain initial and sustained intervention effects multilevel models which accounted for repeated measures and center-level clustering, with a random intercept included at both the child- and kindergarten-center-level. To evaluate initial intervention effects, only T1–T2 data was incorporated in the models. Subsequently a new set of models incorporating data across T1 to T3 were conducted to evaluate whether any early effects were sustained in the short-term (six months), or whether any delayed effects emerging only at T3 were present. All models incorporated a step-up approach; unadjusted models were first tested, followed by adjusted models which included covariates that showed a bivariate correlation with the outcome variable at one time point or more.

A robust intention-to-treat model was assumed for the analyses meaning that all children participating in the study were included in all analyses, regardless of how many RAMSR sessions they attended (in the case of the intervention group); thus, variations in intervention dosage are not accounted for. This approach was used to preserve the benefits of randomization, minimize bias arising from attrition or non-compliance, and provide a conservative and methodologically robust estimate of intervention effects under real-world conditions. Full information likelihood estimation was applied to address missing data, which ranged from 7 to 11% at T1 due to absences during the testing phase, 16–19% at T2 because of children leaving kindergarten or being absent, and 40–60% at T3 resulting from loss of contact, lack of re-consent for follow-up, or non-response from Prep teachers. An analysis of the data indicated that the missingness mechanisms was most likely Missing at Random (MAR; Little et al., 2014), as there was no correlation between missing data at Time 3 and baseline outcome data at Time 1. All children with data for at least one time point on any outcome measure were included in analyses, which were conducted in SPSS Version 29 using a restricted maximum likelihood approach to manage missing data. Estimates of effect sizes for intervention effects are not readily available or able to be interpreted; therefore, we used general linear models to calculate eta-squared effect sizes, which also allows for comparison with prior RAMSR studies (Bentley et al., 2023; Williams et al., 2023). The final dataset contained 213 children.

## 3. Results

### 3.1. Child Outcome Measures: Descriptive and Correlational Data

The bivariate correlations for outcome measures, family demographics, child age, and gender are reported in Table 3. Having a parent who had not completed high school was not significantly associated with any of the outcome measures across timepoints (T1–T3). Children with an Aboriginal background demonstrated higher internalizing behaviors at T3. Female children scored higher in prosocial skills at each time point, higher in sociability at T1 and T3, and lower in externalizing problems at T2 only, compared to males. Children with a non-English speaking background scored lower in prosocial skills at T1 and in sociability across all timepoints. Children with low parental income scored lower in prosocial skills at T1. Children with developmental delay demonstrated lower scores for prosocial and sociability, and higher scores in externalizing and internalizing problems at all time points, compared to children without a developmental delay.

### 3.2. Intervention Effects

In testing each model (one for each of the four outcome variables) for T1 and T2 only, we found no significant intervention effects (see Appendix A); however, there were several delayed intervention effects apparent in models that included all three time points of data collection. The final model results for T1–T3 (adjusted) for each outcome measure are reported in Table 4. There was a significant intervention effect for increased prosocial skills (*p* = 0.043, *ƞ_p_*^2^ = 0.02; controlling for child age, gender, and developmental delay) and reduced externalizing (*p <* 0.01, *ƞ_p_*^2^ = 0.03; controlling for child gender and developmental delay) and internalizing behaviors (*p* = 0.037, *ƞ_p_*^2^ = 0.02; controlling for child developmental delay) for the intervention group compared to the control group, with small effect sizes. There was no significant intervention effect for sociability. These results provide evidence that children who participated in the intervention demonstrated steeper growth trajectories in prosocial skills and reductions in internalizing and externalizing behavior problems.

## 4. Discussion

This study evaluated the impact of a novel Rhythm and Movement for Self-Regulation (RAMSR) intervention on children’s early social and emotional development, documenting benefits related to increased prosocial skills and reduced behavior problems across the transition-to-school period. The RAMSR program translates principles from music psychology, music education, and music therapy into an accessible and engaging classroom-based program that can be delivered by generalist educators with no prior music experience. While findings related to self-regulation and cognitive outcomes from the same trial have been reported previously, showing positive intervention effects for self-regulation, inhibition, and school readiness (Bentley et al., 2023; Williams et al., 2023), to our knowledge, this is the first experimental study to document the social and behavioral benefits of a music engagement program delivered by non-specialists in regular early-childhood classrooms.

Children who participated in the RAMSR intervention had significantly steeper growth in prosocial skills compared to children in the control group. We hypothesize that increases in prosocial skills as observed by school teachers for the intervention group related to children’s experience and practice of interpersonal synchrony supported by rhythmic auditory cueing during RAMSR in kindergarten. Lab-based studies consistently find that moving in time with others stimulates more empathetic and helping behaviors than moving asynchronously (Cirelli, 2018; Rabinowitch & Meltzoff, 2017; Tunçgenç & Cohen, 2018). In early-learning settings, interpersonal synchrony occurs when children move, sing, or play instruments in time with one another and their educators. This shared rhythmic coordination promotes feelings of connectedness and mutual understanding, enabling children to experience being “in sync” with others. The previous literature suggests that such experiences of synchrony are fundamental to the development of social bonding and prosocial behaviors (Kirschner & Tomasello, 2010; Rabinowitch et al., 2024). Within RAMSR, rhythmic movement activities are designed to support these same processes by providing repeated opportunities for children to move together in time, take turns, and share positive emotional experiences. Our findings here reflect prior studies linking group music participation and formal music instruction with prosociality in older children (Good & Russo, 2016; Schellenberg et al., 2015; Wu & Lu, 2021). The findings also reflect prior studies with preschool-aged children, which demonstrate that participation in joint music-making and musical play (compared to non-music conditions) stimulates prosocial behaviors including helping (Beck & Rieser, 2022; Kirschner & Tomasello, 2010).

Children who participated in the RAMSR intervention showed a statistically significant reduction in internalizing and externalizing problems during the transition-to-school period compared to children in the control group. This is an important outcome for this group of children from low socio-economic communities who are at increased risk of poor school transitions, which may mark the beginning of ongoing gaps in achievement and wellbeing trajectories. Behavior problems are closely linked to children’s capacity for emotional and behavioral self-regulation. Our prior studies show that RAMSR has a positive impact on children’s self-regulatory capacities, so it is possible that those early skills supported reductions in teacher-observed behavior problems over time (Bentley et al., 2023; Williams et al., 2023).

Although we expected to see improvements in our key outcomes in the Time 2 data immediately following the intervention, these did not arise until school follow-up. Importantly, the effects were found in data reported by school teachers who were blind to the conditions and were different to the teachers reporting at Time 1 and Time 2, increasing the robustness of our findings. There are a range of reasons why our intervention effects were found only in models including the follow-up data collection point, and therefore can be considered delayed or sleeper effects. As noted above, it is possible that these effects emerged over time as a result of earlier gains in more basic self-regulation skills, reported in the same sample at Time 2 (Williams et al., 2023). The measured outcomes in this paper of prosociality and behavior problems may represent more complex developmental constructs that take time for children to practice and demonstrate, and are indeed mediated by earlier boosts in self-regulation.

No significant difference was identified for general sociability skills. It is possible that for the sociability domain, which reflects children’s ability to initiate and maintain friendships, children must draw on a broader set of social and communicative competencies than those targeted through the RAMSR program. While the program promotes opportunities for interpersonal synchrony and shared rhythmic experiences that are likely to help children feel in sync with their peers, successfully forming and sustaining friendships may additionally depend on language, perspective-taking, and advanced social problem-solving skills that develop over longer timeframes. It is also possible that our measure of sociability tapped relatively stable temperament traits as per the original conception of sociability (Rothbart, 2007), and therefore this construct is less malleable through short-term intervention.

While effect sizes were small, even small gains during early childhood, particularly in the context of the cumulative disadvantage often experienced in low socio-economic communities, can yield meaningful population-level benefits over time. Where programs such as RAMSR can be delivered at scale and embedded within everyday educational practice, early gains in social and behavioral development are likely to provide some protection against poorer learning and wellbeing trajectories common in these communities. RAMSR implementation is relatively cost-effective with a small once-off training fee for teachers (no prior specialist knowledge required) who can then deliver the program year after year with new cohorts of children, boosting scalability. Educational policy and curriculum designers are encouraged to consider way that effective universal approaches such as RAMSR can be integrated in preschool and early-school curriculums, particularly in low socioeconomic communities.

### Limitations and Future Directions

Although this study offers valuable insights into the effectiveness of the RAMSR program, it does have limitations. A major constraint was the substantial participant attrition between the post-intervention phase (T2) and the follow-up assessment in Prep (T3). This was largely due to logistical challenges, including the dispersal of children from eight kindergarten centers to more than 70 different schools, and the extensive re-consent process required from families, schools, teachers, and children. However, we note that our modeling procedure robustly accounted for this missing data. Future research could extend data collection to incorporate parent-reported measures of children’s social and emotional development. Additional measures could be incorporated, including video-based coding of synchronous movements and physiological measures (e.g., heart rate), to capture the degree of movement synchrony during RAMSR sessions among children and educators and explore how these patterns translate to social and emotional outcomes. It should also be noted that the kindergarten teachers who completed the T1 and T2 self-regulation measures were not blind to the conditions. However, at T3 follow up, Prep teachers were completely blind to the conditions, strengthening the validity of the findings.

Finally, the extent to which the findings presented here might be replicated outside of the local Australian context in which the program was developed and tested is yet to be determined. A trial of an adapted version of the program has been completed in Hong Kong with results not yet published. However, early publications suggest that RAMSR was readily adapted to the Cantonese-speaking context given a low use of English language across the program, with two new songs with Cantonese lyrics composed to align with the Hong Kong culture (Bautista et al., 2025a). Further, the Hong Kong version of RAMSR training provided online increased confidence to deliver music and movement activities in 84 intervention teachers compared to 87 control teachers (Ng et al., 2024), and stimulated high levels of RAMSR implementation fidelity (Bautista et al., 2025b). Future studies should examine the effectiveness of the program in terms of children’s developmental outcomes in countries outside of Australia.

## 5. Conclusions

This study demonstrates the effectiveness of the Rhythm and Movement for Self-Regulation (RAMSR) program, which adapts clinical applications of music therapy and evidence from music education into a classroom-based program that educators can implement without prior musical training. The program yielded significant outcomes, including enhanced prosocial skills and reductions in both externalizing and internalizing behaviors. These add to the existing published findings related to self-regulation and executive function and suggest that investment in the future scaling-up of such programs may be a worthwhile endeavor.

## Figures and Tables

**Table 1:** Demographic data for intervention and control groups.

	Whole Sample *N* = 213	Intervention (*n* = 112, 53%)	Control (*n* = 101, 47%)
*M/(SD)*			
Mean child age at T1 (months)	50.54 (4.49)	49.95 (4.30)	51.19 (4.63)
*n* (%)			
Child gender (female)	109 (51%)	61 (54%)	48 (48%)
Parental education: completed high school	143 (67%)	73 (65%)	70 (69%)
Low family income	105 (60%)	56 (50%)	49 (49%)
Child Aboriginal or Torres Strait Islander	35 (17%)	10 (9%)	25 (25%)
Child non-English-speaking background	34 (16%)	21 (19%)	13 (13%)
Child developmental delay	29 (14%)	14 (13%)	15 (16%)

**Table 2:** RAMSR session plan sections and example activities.

Section	Example Activities
Warm-up	Body percussion where children imitate teachers’ movements to varying tempos.
Becoming familiar	A familiar early-childhood song in the local context is chanted or sung by the teacher with associated actions that children copy. Teachers then introduce a ‘brain trick’ by reversing the actions, leaving some out, or changing the tempo.
Moving	An audio track is used to lead children through particular movements where children need to match a movement to the particular sounds or musical style. Start/stop games and additional challenges are applied, including being still during the music and then moving in silence.
Playing to the beat	Either simple rhythm sticks or castanets are used to play along to an audio track including start/stop games, and taking directions on when and what to play using visual cue cards shown by the teacher.
Dancing to the beat	Children complete more complex coordinated gross motor movements to an audio track which often involves remembering a series of moves or completing specific instructed postures during start/stop games.
Calming	An audio track with a recorded script leads children through gentle movements and a focus on breathing and calming, finishing with practicing being still at the end of the session.

**Table 3:** Descriptive statistics and bivariate correlations.

	Variables	1	2	3	4	5	6	7	8	9	10	11	12	13	14	15	16	17	18	19
1	Prosocial T1	--																		
2	Prosocial T2	0.78 **	--																	
3	Prosocial T3	0.67 **	0.76 **	--																
4	Externalizing T1	−0.69 **	−0.58 **	−0.58 **	--															
5	Externalizing T2	−0.60 **	−0.64 **	−0.66 **	0.74 **	--														
6	Externalizing T3	−0.50 **	−0.55 **	−0.74 **	0.66 **	0.79 **	--													
7	Internalizing T1	−0.51 **	−0.48 **	−0.42 **	0.38 **	0.34 **	0.25 **	--												
8	Internalizing T2	−0.44 **	−0.43 **	−0.47 **	0.42 **	0.46 **	0.40 **	0.62 **	--											
9	Internalizing T3	−0.44 **	−0.44 **	−0.55 **	0.36 **	0.41 **	0.53 **	0.48 **	0.68 **	--										
10	Sociability T1	0.71 **	0.53 **	0.42 **	−0.30 **	−0.25 **	−0.16 *	−0.55 **	−0.34 **	−0.36 **	--									
11	Sociability T2	0.64 **	0.66 **	0.44 **	−0.30 **	−0.24 **	−0.13	−0.55 **	−0.48 **	−0.39 **	0.77 **	--								
12	Sociability T3	0.55 **	0.60 **	0.67 **	−0.29 **	−0.31 **	−0.32 **	−0.52 **	−0.49 **	−0.53 **	0.63 **	0.72 **	--							
13	Female	0.19 **	0.16 *	0.21 **	−0.12	−0.16 *	−0.14	−0.16	−0.00	−0.03	0.15 *	0.10	0.20 **	--						
14	Aboriginal or Torres Strait Islander	−0.05	0.02	0.03	−0.05	−0.04	0.08	−0.01	0.12	0.20 **	−0.05	−0.01	0.09	0.01	--					
15	Non-English language	−0.16 *	−0.10	−0.09	0.11	−0.02	−0.05	0.04	−0.07	−0.08	−0.20 **	−0.17 *	−0.16 *	−0.14 *	−0.20 **	--				
16	Parental low income	−0.18 *	−0.10	−0.06	0.11	0.04	0.03	−0.07	0.07	0.08	−0.02	−0.03	0.08	0.14	0.23 **	0.04	--			
17	Parents did not complete high school	−0.02	−0.04	0.08	−0.01	0.06	0.02	0.00	0.01	0.04	−0.01	−0.01	0.08	0.02	0.28 **	−0.05	0.21 **	--		
18	Child developmental delay	−0.30 **	−0.31 **	−0.35 **	0.28 **	0.34 **	0.37 **	0.19 **	0.25 **	0.30 **	−0.23 **	−0.17 *	−0.25 **	−0.08	0.00	−0.13	−0.13	0.11		
19	Child age T1 (months)	0.18 **	0.20 **	0.08	−0.08	−0.04	−0.01	0.02	0.02	0.00	0.16 *	0.15 *	0.10	−0.12	0.02	−0.11	−0.04	0.10		
	Range	1–5	1.2–5	1–5	1–5	1–5	1–5	1–4.2	1–4.8	1–4	1–5	1–5	1–5	NA	NA	NA	NA	NA	40–67	
	Mean	3.51	3.72	1.94	1.94	1.79	1.78	2.20	1.74	1.59	3.43	3.69	3.96						50.54	
	SD	0.95	0.86	0.93	0.93	0.91	0.96	0.66	0.78	0.72	0.90	0.96	1.03						4.50	

Note: * *p* < 0.05; ** *p* < 0.01.

**Table 4:** Adjusted mean differences (95% CI) between control and treatment groups.

	Control T1	Control T2	Control T3	Intervention T1	Intervention T2	Intervention T3	Adjusted Mean Diff	*p* Value	Effect Size *n_p_*^2^
				M (SE)			(95% CI)		
Prosocial	0.61 (0.04)	0.65 (0.04)	0.70 (0.043)	0.48 (0.04)	0.53 (0.04)	0.62 (0.04)	0.03 (−0.50, −0.00)	0.043	0.02
Externalizing	0.26 (0.05)	0.24 (0.05)	0.26 (0.05)	0.40 (0.05)	0.34 (0.05)	0.32 (0.05)	−0.04 (0.14, 0.06)	0.003	0.03
Internalizing	0.32 (0.03)	0.21 (0.03)	0.20 (0.03)	0.37 (0.03)	0.27 (0.03)	0.20 (0.03)	−0.02 (0.00, 0.05)	0.037	0.02
Sociability	0.54 (0.04)	0.63 (0.04)	0.67 (0.04)	0.43 (0.04)	0.48 (0.04)	0.57 (0.04)	0.01 (−0.34, 0.19)	0.559	0.00

## Data Availability

The data that support the findings of this study are openly available in the QUT Research Data Finder at https://researchdatafinder.qut.edu.au accessed 12 January 2026.

## Abbreviations

The following abbreviation is used in this manuscript:

RAMSR	Rhythm and Movement for Self-Regulation

## Appendix A

**Table A1:** Time 1 and Time 2-only adjusted mean differences (95% CI) on outcome measures between control and treatment groups.

	Control T1	Control T2	Intervention T1	Intervention T2	Adjusted Mean Diff	*p* Value	Effect Size *n_p_*^2^
			M (SE)		(95% CI)		
Prosocial	0.57 (0.04)	0.61 (0.04)	0.45 (0.04)	0.50 (0.04)	−0.01(−0.056, 0.036)	0.668	-
Externalizing	0.25 (0.04)	0.24 (0.04)	0.40 (0.04)	0.34 (0.04)	0.04 (−0.01, 0.09)	0.101	-
Internalizing	0.31 (0.03)	0.20 (0.03)	0.37 (0.03)	0.26 (0.03)	−0.01 (−0.05, 0.04)	0.731	-
Sociability	0.53 (0.04)	0.62 (0.05)	0.43 (0.04)	0.48 (0.04)	0.04 (−0.00, 0.08)	0.067	-
